# Generating One Biometric Feature from Another: Faces from Fingerprints

**DOI:** 10.3390/s100504206

**Published:** 2010-04-28

**Authors:** Necla Ozkaya, Seref Sagiroglu

**Affiliations:** 1 Computer Engineering Department, Engineering Faculty, Erciyes University, 38039, Kayseri, Turkey; 2 Computer Engineering Department, Engineering Faculty, Gazi University, 06570 Ankara, Turkey; E-Mail: ss@gazi.edu.tr

**Keywords:** biometrics, fingerprint, face, artificial neural network, intelligent system, Taguchi

## Abstract

This study presents a new approach based on artificial neural networks for generating one biometric feature (faces) from another (only fingerprints). An automatic and intelligent system was designed and developed to analyze the relationships among fingerprints and faces and also to model and to improve the existence of the relationships. The new proposed system is the first study that generates all parts of the face including eyebrows, eyes, nose, mouth, ears and face border from only fingerprints. It is also unique and different from similar studies recently presented in the literature with some superior features. The parameter settings of the system were achieved with the help of Taguchi experimental design technique. The performance and accuracy of the system have been evaluated with 10-fold cross validation technique using qualitative evaluation metrics in addition to the expanded quantitative evaluation metrics. Consequently, the results were presented on the basis of the combination of these objective and subjective metrics for illustrating the qualitative properties of the proposed methods as well as a quantitative evaluation of their performances. Experimental results have shown that one biometric feature can be determined from another. These results have once more indicated that there is a strong relationship between fingerprints and faces.

## 1. Introduction

Biometrics has become more and more important solutions to overcome vulnerabilities of the security systems for people, companies, corporations, institutions and governments. Person identification systems based on biometrics were used in primarily limited applications requiring high security tasks like criminal identification and police work in the beginning, more recently they have been used in a wide range of applications including information security, law enforcement, surveillance, forensics, smart cards, access control, *etc.* because of their reliability, performance and accuracy of identification and verification processes [1–4]. When the biometric literature was reviewed, it was found that there was extensive literature on fingerprint identification and face recognition. The researchers were mostly focused on designing more secure, hybrid, robust and fast systems with high accuracy by developing more effective and efficient techniques, architectures, approaches, sensors and algorithms or their hybrid combinations [1,2].

Generating a biometric feature from another is a challenging research topic. Generating face characteristics from only fingerprints is an especially interesting and attractive idea for applications. It is thought that this might be used in many security applications. This challenging topic of generating face parts from only fingerprints has been recently introduced for the first time by the authors in series of papers [5–13]. The relationships among biometric features of the faces and fingerprints (Fs&Fs) were experimentally shown in various studies covering the generation of:

- face borders [5],
- face contours, including face border and ears [6],
- face models, including eyebrows, eyes and mouth [7],
- inner face masks including eyes, nose and mouth [8],
- face parts, including eyes, nose, mouth and ears [9],
- face models including eyes, nose, mouth, ears and face border [10],
- face parts, including eyebrows, eyes, nose, mouth and ears [11],
- only eyes [12],
- face parts, including eyebrows, eyes and nose [13],
- face features, including eyes, nose and mouth [14] and
- face shapes, including eyes, mouth and face border [15].

In these studies, face parts are predicted from only fingerprints without any need of face information or images. The studies have experimentally demonstrated that there are close relationships among faces and fingerprints.

Although various feature sets of faces and fingerprints, different parameter settings and reference points were used to achieve the tasks with high accuracy from only fingerprints, obtaining the face parts including the inner face parts with eyebrows and face borders with ears has not been studied up to now. In order to achieve the generation task automatically with high accuracy, a complete system was developed. This system combines all the other recent studies introduced in the literature and provides more complex and specific solutions for generating whole face features from fingerprints. In order to improve the performance of the proposed study, Taguchi experimental design technique was also used to determine best parameters of artificial neural network (ANN) models used in this generation. In order to evaluate and demonstrate the results more precisely, 10-fold cross validation technique with both quantitative (objective) evaluation metrics and expanded qualitative (subjective) evaluation metrics were used. So the performance and accuracy were demonstrated in a more reliable way with a limited database in comparison to the previous studies.

The paper is organized as follows. Section 2 reviews the background information on biometrics, automatic fingerprint identification and verification systems (AFIVSs), and face recognition systems (FRSs). Section 3 briefly introduces ANNs. Section 4 presents the motivations of this study as well as investigates the previous works about relationships among fingerprints and faces. Section 5 describes the evaluation methods. Section 6 presents the novelty of the proposed system including basic notations, definitions and various steps of the present method, the intelligent biometric feature prediction system (IBFPS). The experiments including numerical and graphical results of IBFPS are depicted in Section 7. Finally, the proposed work is concluded and discussed in Section 8.

## 2. Background of Biometric Systems

Biometric features covering physical or behavioral characteristics including fingerprint, face, ear, hand geometry, voice, retina, iris recognition, *etc.* are *peculiar* to the individual, *reliable* as far as not being transferable easily and *invariant* during the life time [1]. Typical biometric systems include enrollment, identification, verification, recognition, screening or classification processes. The steps in system tasks are as follows: biometric data acquisition, feature extraction, registration, matching, making decision and evaluation. Biometric data were obtained from people with the help of a camera-like-device for the faces and fingerprint scanner for the fingerprints, *etc.* In general, after data acquisition processes, the digital representation of the biometric data of the people were obtained in the digital platform. Feature extraction processes were applied to this digital form of the biometric features and feature sets were registered to the biometric system database. When a user wants to authenticate him/her self to the system, a fresh biometric feature was acquired, the same feature extraction algorithm is applied, and the extracted feature set is compared to the template in the database. If these feature sets of the input and the template biometric features are sufficiently similar according to the matching criteria, the user’s final decision was taken and the user was authenticated at the end of the matching process [3,14].

Data acquisition, verification, identification and screening phases are the main types of biometric based systems [4]. The types are summarized as:

*Type I:* The biometric data acquisition phase is the first step of the other three phases. Enrollment, classification and recording of the biometric features are achieved in this phase.
*Type II:* The verification phase is the most commonly used biometric system mode in the social life like person identification systems in physical access control, computer network logon or electronic data security [2,4]. In that phase an individual’s identity is usually achieved via a user name, an identification number, a magnetic card, a smart card, *etc.* At the end of the verification phase, the submitted claim of the identity is either rejected or accepted [1].
*Type III:* The identification phase is commonly used in applications requiring high security tasks like criminal identification and police work. In that phase, the system tries to recognize an individual’s identity with using just his or her biometric feature. The system fails if the person is an undefined person in the system database. In that case, the output of the system is a combination list of identities and the scores indicates the similarity among two biometric features [15]. According to some pre-defined rules about similarity measures, the system decision was produced in this phase.
*Type IV:* The screening phase is like the identification phase. The results of determination whether a person belongs to a watch list of identities or not is displayed in this phase. Security at airports, public events and other surveillance applications are some of the screening examples [4,16].

**Figure 1.**
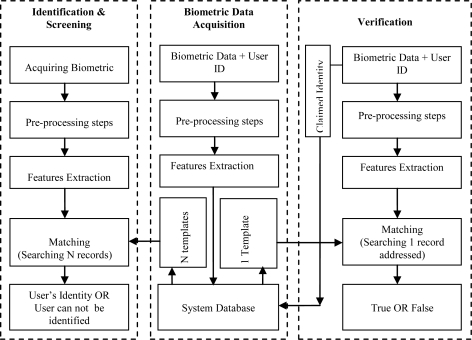
A typical biometric system.

A typical biometric system is given in Figure 1. The processes in the system are achieved according to the arrows illustrated in the figure depending on the application status.

These sort of biometric recognition systems make people, systems or information safer by reducing the fraud and leading to user convenience [4]. Two of most popular biometric features used in the biometric based authentication systems are fingerprints and faces. Fingerprints based biometric systems are called AFIVSs and faces based biometric systems are called FRSs.

Fingerprints are unique patterns on the surface of the fingers. Fingerprints represent the people with high accuracy because of having natural identity throughout the life of which are not forgotten anywhere or not be lost easily. They were reliably and widely used to identify the people for a century due to its uniqueness, immutability and reliability [17].

**Figure 2.**
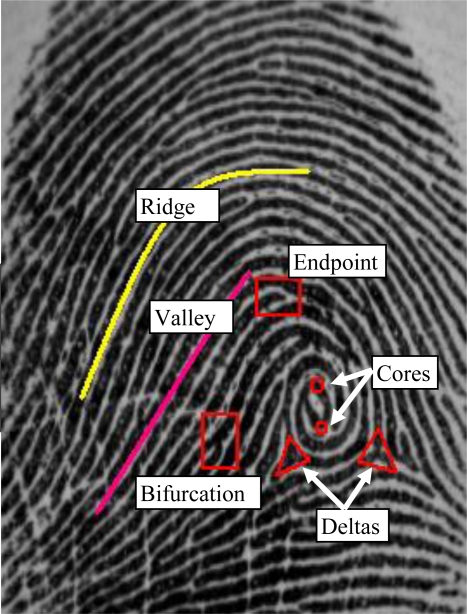
Ridge-valley structure and features of a fingerprint.

In AFIVSs, ridge-valley structure of the fingerprint pattern, core and delta points called singular points, end points and bifurcations called minutiaes are used for identifying an individual. These structures are given in Figure 2. Many approaches to AFIVSs have been presented in the literature [1,2,15,17–30]. The AFIVSs might be broadly classified as being *minutiae-based, correlation-based* and *image-based* systems [18]. A good survey about these systems was given in the reference [1]. *The minutiae-based approaches* rely on the comparisons for similarities and differences of the local ridge attributes and their relationships to make a personal identification [19–21]. They attempt to align two sets of minutiae from two fingerprints and count the total number of matched minutiae [4]. If a minutiae and its parameters are computed relative to the singular points which are highly stable, rotation, translation and scale invariant, the minutiae will then become rotational, translational and scale invariant [15,22–24]. Core points are the points where the innermost ridge loops are at their steepest. Delta points are the points from which three patterns deviate [23,25,26]. The general methods to detect the singular points are Poincare-based [27], intersection-based [23] or filter-based [28] methods.

**Figure 3.**
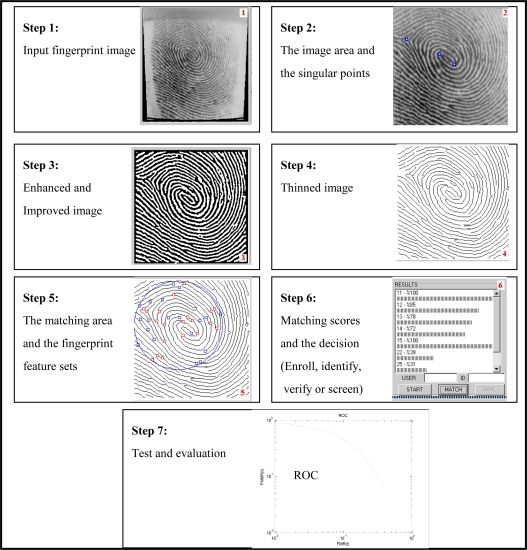
Main operational steps of minutiae-based AFIVSs [29].

Main steps of the operations in the minutiae-based AFIVSs are summarized as: selecting the image area; detecting the singular points; enhancing, improving and thinning the fingerprint image; extracting the minutiae points and calculating their parameters; eliminating the false minutiae sets; properly representing the fingerprint images with their feature sets; recording the feature sets into a database; matching the feature sets; and, testing and evaluating the system [29]. The steps and their results are given in Figure 3, respectively. Although the performance of the minutiae-based techniques relies on the accuracy of all these steps, the feature extraction and the use of sophisticated matching techniques to compare two minutiae sets are often more effective on the performance.

Global patterns of the ridges and valleys are compared to determine if the two fingerprints are aligned in the correlation-based AFIVSs. The template and query fingerprint images are spatially correlated to estimate the degree of similarity between them. The performance of correlation-based techniques is affected by non-linear distortions and noises in the image. In general, it has been observed that minutiae-based techniques perform better than correlation-based ones [30]. The decision is made using the features that are directly extracted from the raw image in the image-based approaches that might be the only viable choice when image quality is too low to allow reliable minutiae extraction [18].

**Figure 4.**
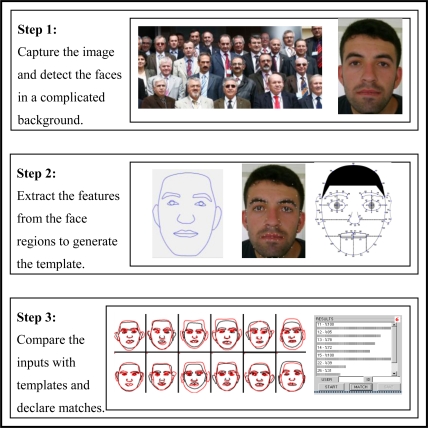
Main processes of face processing for fingerprint to face task system.

Faces are probably the most highly accepted and user-friendly characteristics in the field of biometrics. Face recognition is an attractive and active research area with several applications ranging from static to dynamic [19]. In general, a FRS consists of three main steps covering detection of the faces in a complicated background, extraction of the features from the face regions and localization of the faces and finally recognition tasks [31]. The steps used in face processing in fingerprint to face task are illustrated in Figure 4.

Face recognition process is really complex and difficult due to numerous factors affecting the appearance of an individual’s facial features such as 3D pose, facial expression, hair style, make-up, *etc.* In addition to these varying factors, lighting, background, scale, noise and face occlusion, and many other possible factors make these tasks even more challenging [31]. The most popular approaches to face recognition are based on each location and shape of the facial attributes including eyes, eyebrows, nose, lips and chin and their spatial relationships or the overall analysis of the face image representing a face as a weighted combination of a number of canonical faces [4,32]. Many effective and robust methods for the face recognition have been also proposed [2,19,31–35]. The methods are categorized in four groups as follows [34]: human knowledge of what constitutes a typical face was encoded in the knowledge-based methods. Structural features that exist even when the pose, viewpoint or lighting conditions vary to locate faces were aimed to find in the feature invariant methods. Several standard patterns of a face were used to describe the face as a whole or the facial features separately in template matching based methods. Finally, appearance-based methods operate directly on images or appearances of the face objects and process the images as two-dimensional holistic patterns.

As explained earlier, processing fingerprints and faces are really difficult, complex and time consuming tasks. Many approaches, techniques and algorithms have been used for face recognition, fingerprint recognition and their sub steps. It is very clear from the explanations that dealing with generating faces from fingerprints are really more difficult tasks. Because of the tasks to be achieved in this article, faces, fingerprints, pre and post processing of them, applying many methods, implementing them in training and test procedures, analyzing them with different metrics, and representing the outputs in visual platform, *etc.* have made the prediction task more difficult.

## 3. Artificial Neural Networks

ANNs are biologically inspired intelligent techniques to solve many problems [36–40]. Learning, generalization, less data requirement, fast computation, ease of implementation and software and hardware availability features have made ANNs very attractive for many applications [36].There has been a growing research interest in security and recognition applications based on intelligent techniques and especially ANNs which are also very popular in biometric-based applications [5–13,29,34,35,37–40]. Multilayered perceptron (MLP) is one of the most popular ANN architectures and can be trained with various learning algorithms. Because an MLP structure can be trained by many learning algorithms, it has been successfully applied to a variety of problems in the literature [36].

**Figure 5.**
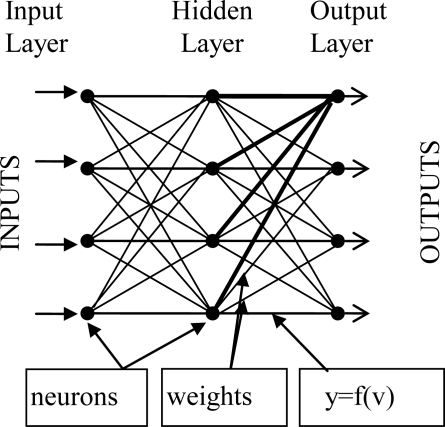
General Form of the MLP.

The MLP structure consists of three layers: input, output and hidden layers. One or more hidden layers might be used. The neurons in the input layer can be treated as buffers and distribute input signal to the neurons in the hidden layer. The output of each neuron in the hidden layer is obtained from the sum of the multiplication of all input signals and weights that follow all these input signals. The sum can be calculated as a function. This function can be a simple threshold function, a hyperbolic tangent or a sigmoid function. The outputs of the neurons in other layers are calculated in the same way. The function can be a simple threshold function, a hyperbolic tangent or a sigmoid function. The outputs of the neurons in other layers are calculated in the same way. The weights are adapted with the help of a learning algorithm according to the errors occurring in the calculation. The errors can be computed by subtracting the ANN outputs from the desired outputs. MLPs might be trained with many different learning algorithms [36]. A general form of the MLP is given in Figure 5.

In this study, the MLP based model structure having single hidden layer was used to model the relationships and to generate the faces. The MLP models were trained with the conjugate gradient algorithm updating weight and bias values according to the conjugate gradient with Powell-Beale restarts (CGB) [41].

## 4. Motivation of the Proposed Approach

It is especially difficult to believe that there is a relationship between biometric features because of their characteristics such as their uniqueness. This research was difficult and challenging. As an initial step, biological and physiological evidences regarding the relationships among biometric features to support this study were investigated. The evidences and observations given below help us to believe that it is worth investigating the relationship among fingerprints and faces. These are given below:

1. It is known that the phenotype of the biological organism is uniquely determined by the interaction of a specific genotype and a specific environment [42]. Physical appearances of faces and fingerprints are also a part of an individual’s phenotype. In the case of fingerprints, the genes determine the general characteristics of the pattern [42]. In dermatoglyphics studies, the maximum generic difference between fingerprints has been found among individuals of different races. Unrelated persons of the same race have very little generic similarity in their fingerprints, parent and child have some generic similarity as they share half of the genes, siblings have more similarity and the maximum generic similarity is observed in identical twins, which is the closest genetic relationship [43].
2. Some of the scientists in biometrics have focused on analyzing the similarities in fingerprint minutiae patterns in identical twin fingers [42]. They absolutely confirmed that the identical twin fingerprints have a large class correlation. In addition to this class correlation, correlation based on other generic attributes of the fingerprint such as ridge count, ridge width, ridge separation, and ridge depth was also found to be significant in identical twins [42].
3. In the case of faces, the situation is very similar with the circumstances of fingerprints. The maximum generic similarity is observed in the identical twins, which is the closest genetic relationship [43].
4. A number of studies have especially focused on analyzing the significant correlation among faces and fingerprints of identical twins [42,44–46]. The large correlation among biometrics of identical twins was repeatedly indicated in the literature by declaring that identical twins would cause vulnerability problems in security applications [47]. The similarity measure of identical twin fingerprints is reported as 95% [47]. The reasons of this high degree similarity measure were explained in some studies as follow:
  - Identical twins have exactly identical DNA except for the generally undetectable micro mutations that begin as soon as the cell starts dividing [46].
  - Fingerprints of identical twins start their development from the same DNA, so they show considerable generic similarity [48].

**Figure 6.**
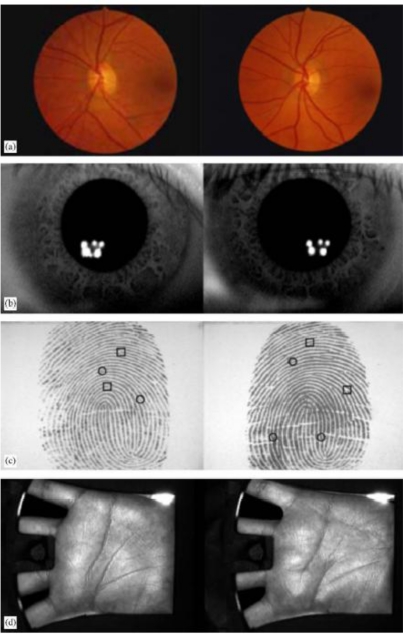
Different biometric features of identical twins [45]. (a) Retina, (b) Iris, (c) Fingerprint and (d) Palm print.

**Figure 7.**
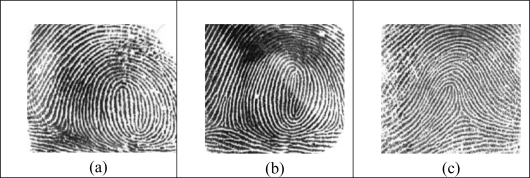
Fingerprints of identical twins (a, b), and fingerprint of another person (c) [46].

**Figure 8.**
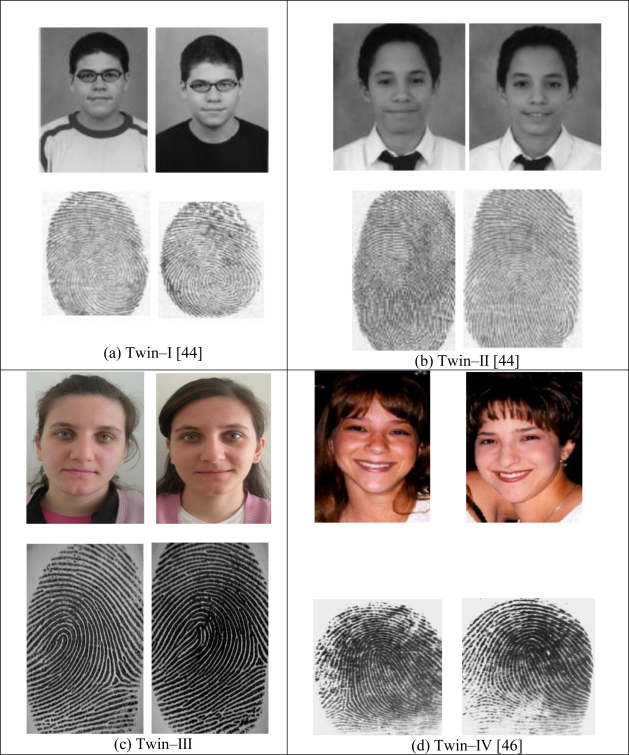
Fingerprints and faces for identical twins.

The similarity among biometric features of identical twins was given in Figure 6. Fingerprints of identical twins and fingerprint of another person were given in Figure 7 [46]. The high degree of similarity in fingerprints or faces of identical twins is demonstrated in Figure 8.

## 5. Previous Work on Relationships among Fingerprints and Faces

In the light of explanations in the previous section, identical twins have strong similarities in both fingerprints and faces. Increasing and decreasing directions of these similarities are also the same among the people. Consequently, this similarity supports the idea that there might be some relationships among fingerprints and faces. The results reported by the authors have been also experimentally shown that relationships among fingerprints and faces exist [5–13].

In the studies [5–13], relationships among fingerprint and face parts were investigated and various face parts were tried to be predicted from just fingerprints step by step from simple to complex. At the beginning of the processes, authors have tried to generate only face borders [5], only eyes [13] and face contours [6] from just fingerprints. In further steps of the process, the ANN structures were improved, trained and tested to predict static face parts [7,8,12]. After these studies, ANN structures used in predicting process were advanced owing to the experiences of the authors and more complex face parts would be generated with high accuracy [9–11]. Finally, this study introduces for the first time the most complex representation of the relationships among fingerprints and faces. The studies [5–13] presented the experimental results in different platforms such as traditional evaluation platform, numerical evaluation platform and finally a visual evaluation platform. However it should be noted that because of having limited data sets covering 120 people in those studies, 10-fold cross-validation should be applied to illustrate the performance of the system. Randomly selected train-test data sets are no longer appropriate to characterize the performance of the system. It can lead into error in evaluating the performance of the system by causing imperfect comments on the results. In 10-fold cross validation process, the database was randomly divided into 10 different data group sets covering 90% of all data set in training and the rest 10% in test data sets for each fold. The proposed system was trained and tested with these ten different training-test data sets. After ten different trainings, 10 test processes were then followed. Accuracy and performance of the ANN models for each fold were computed according to the appropriate evaluation metrics covering expanded quantitative and qualitative metrics.

The ANN structures of previous studies were designed and reconfigured with randomly selected or experimentally obtained parameters. It is well known that finding appropriate parameters depending on applications is very difficult. It takes time and suitable parameters are established with the help of trails and errors. To do it systematically, as mentioned before, this study also presents obtaining best ANN parameters like numbers of the layers, numbers of the inputs, training algorithms and activation functions with the help of Taguchi experimental design technique.

In the previous studies [5–13], performance and accuracy of the proposed model are evaluated by quantitative metrics and/or human assessment presented in a graphical form. In this paper, both the quantitative measures (*i.e.*, objective) carried out automatically by computers expanding the metrics available in the literature and the qualitative (subjective) evaluation perceived by observation were taken into account. Next section describes these quantitative and qualitative evaluation metrics.

## 6. Evaluation metrics

To generate more accurate face features from fingerprints without having any information about faces is successfully achieved and introduced in this study. It needs to be emphasized that evaluating results was an important, critical and difficult part in this study. There were not certain criteria to elaborate the results precisely. For doing that, the success and reliability of the proposed system having proper metrics in achieving face parts from only fingerprints must be clearly illustrated.

The traditional metrics of an ordinary biometric system like FMR-FNMR representation and ROC curve are no longer appropriate to characterize the performance of the system because of the proposed system is not an ordinary biometric-based recognition system. In this study, more test procedure and performance metrics covering combination of the quantitative and qualitative measures are introduced for better evaluations. The details of these metrics are explained in the following subsections.

### 6.1. Quantitative Evaluation Metrics

These metrics are briefly introduced in the following subsections.

#### 6.1.1. FMR-FNMR Curve and The ROC Curve

FMR-FNMR and ROC curves are commonly used as evaluation metrics for biometric based recognition systems. The curves and determination procedure were detailed in [1]. The null (H_o_) and alternate (H_1_) hypotheses for the biometric verification problem and associated decisions according to these hypotheses were given in Table 1 and Table 2, respectively. If “T” is stored as a biometric template of a person and “I” is the acquired input of a biometric feature, the hypotheses for biometric verification are written for H_o_:I≠T input and template do not come from the same person and H_1_: I=T input and template come from the same person.

In general, two types of errors are encountered in a typical biometric verification system: mistaking biometric measurements from two different fingers being the same finger (false match) and mistaking two biometric measurements for the same finger being two different fingers (false non-match). These errors are given in Table 3 for Type I and Type II, respectively. The verification involves matching T and I using a similarity measure s(T,I). If the matching score s(T,I) is less than the system threshold t, then decide D_o_, else decide D_1_. To evaluate the system, it must be collected the scores generated from a number of fingerprint pairs from the same finger (the distribution p(s|H_1_ = true) of such scores is traditionally called genuine distribution), and scores generated from a number of fingerprint pairs from different fingers (the distribution p(s|H_o_ = true) of such scores is traditionally called impostor distribution). FMR is the probability of Type I error and could be defined as the percentage of impostor pairs whose matching score greater than or equal to t, and FNMR is the probability of Type II error and could be defined as the percentage of genuine pairs whose matching score is less than t.

Among FMR and FNMR, there is a strict tradeoff. If t is decreased to make the system more tolerant with respect to input variations and noise, then FMR increases; *vice versa*, if t is raised to make the system more secure, then FNMR increases accordingly. So the system performance was reported at all operating points (threshold, t) in ROC curves by plotting FNMR as a function of FMR [1].

#### 6.1.2. Mean Squared Error (MSE) and Sum Squared Error (SSE)

MSE and SSE are the metrics to quantify the amount by which an estimator differs from the true value of the quantity being estimated. These metrics were used for evaluation of the performance and accuracy of the systems that were investigating the relationships among fingerprints and faces in the literature [5]–[13]. MSE is to measure the average of the square of the error. SSE is the sum of squared predicted values in a standard regression model. In general, the less the SSE, the better the model performs in its estimation. MSE and SSE were given in Equations (1) and (2), respectively. In the Equations, *n* is the number of the test people, *O_i_* is the output of the system and *D_i_* is the desired value of *O_i_*:

(1) $$\mathrm{MSE}=\frac{1}{n}\sum_{i=1}^{n}{\left(D_{i}-O_{i}\right)}^{2}$$

(2) $$\mathrm{SSE}=\sum_{i=1}^{n}{\left(D_{i}-O_{i}\right)}^{2}$$

#### 6.1.3. Absolute Percentage Error (APE) and Mean APE (MAPE)

APE is the measure of accuracy in a fitted time series value. It usually expresses accuracy as a percentage [50]. APE is also commonly used as an evaluation metric in the similar studies aimed to investigate among fingerprints and faces in the literature [5]–[13]. These metrics were given in Equations (3) and (4). In the equations, *n* is the number of the test people, *O_i_* is the output of the system and *D_i_* is the desired value of *O_i_*:

(3) $$\mathrm{APE}=\sum_{i=1}^{n}\frac{\left|D_{i}-O_{i}\right|}{D_{i}}$$

(4) $$\mathrm{MAPE}=\frac{1}{n}\sum_{i=1}^{n}\frac{\left|D_{i}-O_{i}\right|}{D_{i}}$$

#### 6.1.4. Mean Absolute Error (MAE)

MAE is a quantity used to measure generations or predictions how they are close to the eventual outcomes. This metric was used in this study at first. It should be noted that, this metric was linked appropriately with the application proposed in this paper. As the name suggests, MAE is an average of the absolute errors. It is calculated average of the absolute errors per each coordinate of the feature sets of the faces in the proposed study. The formulation of MAE is given in Equation (5). In the equation, *O_i_* is the output of the ANN, *D_i_* is the desired value of the *O_i_* and *e_i_* = *D_i_* *− O_i_*:

(5) $$\mathrm{MAE}=\frac{1}{n}\sum_{i=1}^{n}\left|D_{i}-O_{i}\right|=\frac{1}{n}\sum_{i=1}^{n}\left|e_{i}\right|$$

### 6.2. Qualitative Evaluation Metrics

In previous studies [5–13], quantitative evaluation platforms were prepared to help the researchers determine whether the obtained results are similar to their desired values or not. In this study, in addition to that, a qualitative analysis was carried out in order to determine whether the obtained results are similar to their desired values, how much the results are close to their desired values and how accurately the system performs the task. Although the quantitative metrics indicate the system performance clearly in the numerical manner, they do not provide any information about the perceived visual quality of the results. Accordingly, a psychophysical experiment was designed and carried out below.

The aim of this qualitative analysis was to determine which quality of results the system produces imagery with the highest perceived results quality by human observers. Qualitative assessment method applied to this study was explained below.

In order to obtain an objective qualitative assessment of the results, a standard psychophysical rank-ordering paradigm [51,52] was employed to modify the paradigm for our study. Essentially, this paradigm consisted of presenting the participants with the results and asking each participant to rank order of each of those results based on their “qualities” by assigning each of the results in a numerical value. Specifically, in this study the test results for each fold were presented to the participants by asking each participant to the degree of the results in a numerical value from 1 to 5. The meanings of the numerical values are given below:

1. the results are very different from the desired values, the system failed.
2. the results are a bit similar to the desired values, but the system cannot be accepted as successful.
3. the results are similar to the desired values, the system success is average.
4. the results are very similar to the desired values, the system is above average.
5. the results are nearly the same or the same with the desired values, the system is very successful.

Before starting the experiments each participant was asked to read standardized instructions explained the task clearly. All participants were allowed to ask questions regarding the task before beginning the experiments. At the beginning of the experiments, for each trial, twelve results for each 10-fold cross validation were simultaneously displayed. At the end of each checking process, he or she gives a mark for the test results of each fold. At the end of this part of the evaluation, each participant checks all test results of the 10-fold cross validation containing 120 test people and gives a mark for each fold to evaluate the results if face prediction is successfully achieved or not.

## 7. The Proposed System: Intelligent Biometric Feature Prediction System (IBFPS)

In order to achieve the task of prediction, a proposed system called IBFPS was developed and implemented. The new approach successfully generates total face features containing all of the face parts including eyebrows, eyes, nose, mouth and face contours including face border and ears from only fingerprints without having any information about faces in this study. In addition, the relationships among Fs&Fs are also analyzed and discussed in more details with the help of different evaluations criteria.

Assume that this relationship among faces and fingerprints can be mathematically represented as:

(6) y=H(x)

where *y* is a vector indicating the feature set of the face model and its parameters achieved from a person, *x* is a vector representing the feature set of the fingerprint acquired from the same person, *H(.)* is a highly nonlinear system approximating *y* onto *x*. In this study, *H(.)* is approximated to a model to generate the relationship among Fs&Fs with the help of ANN models.

The proposed system is based on MLP-ANN model having the best parameters with the help of Taguchi experimental design technique [53–55]. MLPs were trained with the binary input vectors and the corresponding output vectors with different parameter levels based on Mean Square Errors (MSEs) and Absolute Percentage Errors (APEs).

In order to determine the best parameters of MLP-ANN structure, L-16 (8^**^1 2^**^3) Taguchi experiment is designed. Taguchi design factors and factor levels were given in Table 4. Training algorithms, the numbers of layers, the numbers of inputs and the transfer functions were main Taguchi design factors and 8, 2, 2 and 2 to be considered as factor levels, respectively.

MLP-ANN training algorithms considered and used in this work were Powell-Beale conjugate gradient back propagation (CGB), Fletcher-Powell conjugate gradient (CGF), Polak-Ribiere conjugate gradient (CGP), Gradient Descent (GD), Gradient Descent with adaptive learning coefficients (GDA), One Step Secant (OSS), GDA with momentum and adaptive learning coefficients (GDAM) and scaled conjugate gradient (SCG) [56].

In this study, the numbers of layers were set to 3 and 4, the numbers of inputs were 200 and 300. Hyperbolic Tangent (HT) and Sigmoid Function (SF) activation functions were considered and used in MLP-ANN structures.

In Taguchi design, best parameters of MLP-ANNs were determined according to MSEs. Main effect plots were taken into considerations while analyzing the effects of parameters on the response factor. These plots might help to understand and to compare the changes in the level means and to indicate the influence of effective factors more precisely. According to these plots, training algorithms had the largest main effect on MSE. The numbers of layers in MLP-ANN structure, and transfer functions were also considerably effective. MSEs were not mainly affected by the numbers of inputs. Finally it can be clearly said that considering the main effect plots, MSEs will get smaller if the parameter settings given in Table 5 were followed.

**Figure 9.**
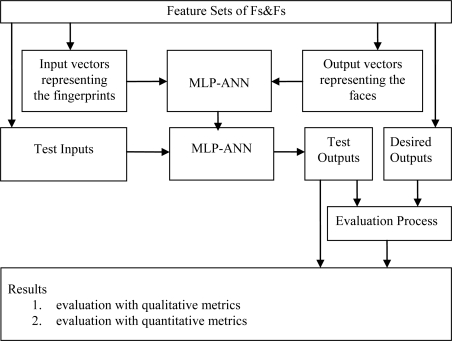
The block diagram of the MLP NN structure.

After the ANN structure and its training parameters were determined to achieve accurate solutions, the training processes were started with applying the fingerprint and face feature sets of the people to the system as inputs and outputs, respectively. The sizes of input and output vectors were also 300 and 176, respectively. The system achieves the training processes with these feature sets according to the learning algorithm and the ANN parameters which were obtained from Taguchi design method. Even if the feature sets of Fs&Fs were required in training, only fingerprint feature sets were used in test. It should be emphasized that these fingerprints used in test were totally unknown biometric data to the system. The outputs of the system for the unknown test data indicate the accuracy of the system. The success and reliability of the system must be clearly shown by evaluating the ANN outputs against the proper metrics in achieving face parts from fingerprints. The block diagram of the MLP-ANN used in this work is given in Figure 9.

According to the best parameters obtained from Taguchi method, the MLP-ANN models were trained with a conjugate gradient algorithm that updates weight and bias values according to the conjugate gradient back propagation with Powell-Beale restarts (CGB). The CGB is a network training algorithm that updates weight and bias values according to the CGB algorithm [56]. Conjugate gradient algorithms (CGAs) execute very effective search in the conjugate gradient direction. Generally, a learning rate is used to determine the length of the step size. For all CGAs, the search direction will be periodically reset to the negative of the gradient. The standard reset point occurs when the number of iterations is equal to the number of network parameters (weights and biases), but there are other reset methods that can improve the efficiency of training [57]. One such reset method was proposed by Powell [41], based on an earlier version proposed by Beale [58].

In principle, feed forward neural networks for non-linear system identification can use all CGAs. In the first iteration, the CGAs start out by searching in the steepest descent direction that was given in Equation (7):

(7) $$p_{0}=-g_{0}$$

In the equation, *p_o_* and *g_o_* are the search vector and gradient, respectively. Consider *x_k_* is the estimate of the minimum at the start of the *k*-th iteration. The *k*-th iteration then consists of the computation of search vector *p_k_* from which new estimate *x_k+1_* is obtained. It is given in Equation (8):

(8) $$x_{k+1}=x_{k}+\alpha_{k}p_{k}$$

In the equation, α*_k_* is previous knowledge based upon the theory of the method or obtained by linear search. The next search direction is determined so that it is conjugate to previous search directions. Combining the new steepest descent direction with the previous search direction is the general way for determining the new search direction. It is given in Equation (9). In the equation, β*_k_* is a positive scalar and the various versions of gradient are distinguished by the manner constant β*_k_* is computed [59]:

(9) $$p_{k}=-g_{k}+\beta_{k}p_{k-1}$$

Periodically resetting the search direction to the negative of the gradient improves the CGAs. Since Powell-Beale procedure is ineffective, a restarting method that does not abandon the second derivative information is needed. According to Powell-Beale technique it will restart if there is very little orthogonality left between the current gradient and the previous gradient. This is tested with the inequality given in Equation (10). If this condition is satisfied, the search direction is reset to the negative of the gradient:

(10) $$\left|g_{k-1}^{T}g_{k}\right|\geq 0.2{\left\|g_{k}\right\|}^{2}$$

**Figure 10.**
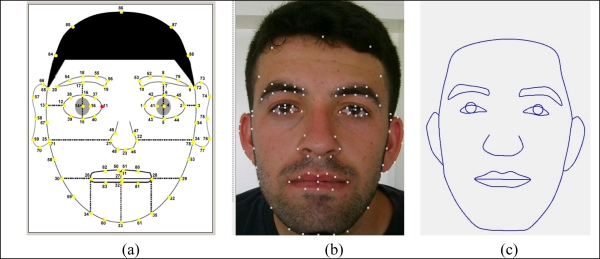
Face reference points a) on the template, b) on a real face image from the database, c) re-construction of the face model from the reference points.

The inputs and outputs of the system were digital representations of fingerprints and faces of the people, respectively. The feature vectors of the fingerprints obtained from a commercially available software development kit contain the local and global feature sets of the fingerprints including singularities, minutiae points and their parameters [60]. Detailed explanation of the feature extracting algorithms, extensive information of fingerprint feature sets and their storage format were given in the reference [60]. These discriminative data represent the people with high accuracy. The outputs were the feature vectors of the faces obtained from a feature-based face feature extraction algorithm that was borrowed from Cox *et al.* [61] and fundamentally modified and adapted to this application. Increasing the number of the reference points 35 to 88 helped to represent the faces more accurately and sensitively. Face feature sets were also shaped from Cartesian coordinates of the face model reference points not distances or average measures as given in the reference [61]. It was also observed that feature sets contain enough information about faces for getting them again with high accuracy. The face reference points on the template, on the face image of a person from our database and re-construction of the face model from the reference points were given in Figure 10.

A flexible design environment for the face model re-construction converting the ANN outputs and/or the desired outputs to visual face models was also included in the software developed. Indeed, it basically transformed the reference points of the face models to the lines. The software is capable of plotting the results of actual and/or calculated values of the same face in the same platform or in different platforms. It also illustrates the ANN results on the real face images. So, the face model re-construction handles an important task for the system by creating two different visual evaluation platforms. This re-construction process enables users to achieve the qualitative evaluation processes easily, efficiently and automatically with the support of the developed useful graphical interface.

**Figure 11.**
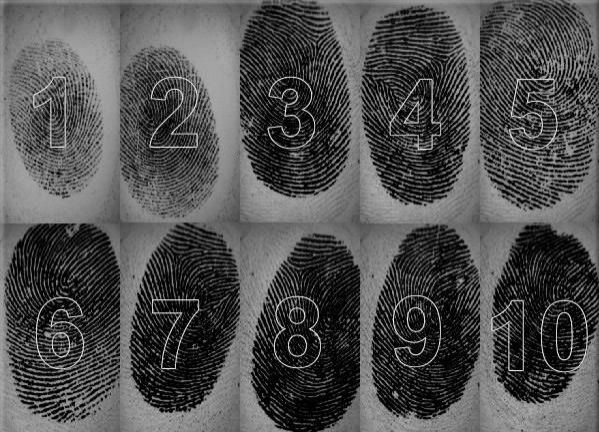
Ten fingerprint images of an individual from our database (from “1” to “10”, from the left to the right, respectively).

**Figure 12.**
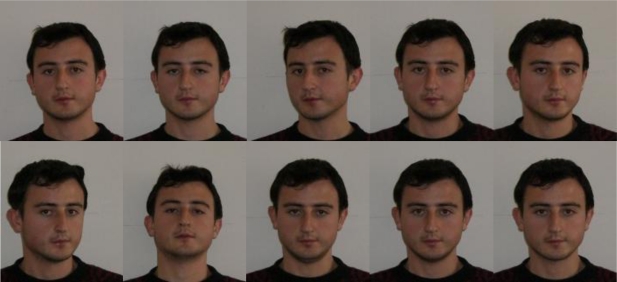
Face images captured from different angles from an individual.

At the beginning of the experiment, an enrollment procedure was followed for collecting the biometric data from the people. This enrollment procedure helps to store fingerprint and face biometrics of individuals into the biometric system database. During this process a real multimodal database belonging to 120 persons was established. Ten fingerprints of each individual were scanned with a fingerprint scanner, and a 10 face image having different angles were also taken from the people using a digital camera. A set of examples including fingerprints and faces of an individual were given in Figure 11 and Figure 12, respectively. Only one frontal face image and one fingerprint belonging to the right hand index finger for each person were used in this study.

The software developed achieves all the tasks of the system from the enrollment step to evaluation step completely. It is expected that generating faces from fingerprints without having any priori knowledge about faces will find considerable attention in science and technology of biometrics, security and industrial applications.

As mentioned earlier, evaluating this system is very critical from the point of being a pioneering study claiming to generate the facial parts including the inner face parts with eyebrows and face contour with ears from only fingerprints. So, the success and reliability of the system must be clearly depicted. In that case, test processes in this article were mainly divided into two main parts: qualitative and quantitative evaluation platforms.

## 8. Experimental Results

In order to achieve the experiments effectively, automatically and easily, a software platform covering Figures 3, 4 and 5 was developed.

In order to generate faces from only fingerprints, the following experiments were performed as:

1. Read fingerprints and faces from database
2. Obtain the feature sets of fingerprints and faces.
3. Establish a network configuration for training
4. Find optimum parameters with the help of Taguchi method.
5. Train the network with selected parameters.
6. Save the results for further uses.
7. Test the system against to the proper evaluation metrics.
8. Test the system performance based on 10-fold cross validation technique.
9. Investigate whether the quantitative (objective) evaluation results are consistent with qualitative (subjective) evaluations based on human perceptual assessment.

Previous experiments on predicting faces from fingerprints [5–13] have shown that the relationship between fingerprints and faces can be also achieved with high accuracy. In the current experiments, an automatic and intelligent system based on artificial neural network is designed to generate the faces of people from their fingerprints only. The proposed study has some advantages on the previous studies in the literature. These features are given below as:

1. All face parts including eyebrows, eyes, nose, mouth, face border and ears were successfully predicted in this study for the first time.
2. The optimal parameters of ANN model parameters were determined with the help of Taguchi experimental design technique.
3. Qualitative evaluation procedure was followed in addition to the quantitative evaluation procedure with some extra quantitative metrics.
4. 10-fold cross validation technique was applied to analyze and to evaluate the performance and the accuracy of the system more precisely.

Producing the face models as close as possible to the real one is the most critical part of the system in this study. In order to evaluate the performance of the developed system effectively, test experiments were mainly focused on two qualitative and quantitative evaluation platforms: a 10-fold cross-validation method was followed, as mentioned earlier. The results of the system were tested against to these evaluation metrics.

**Figure 13.**
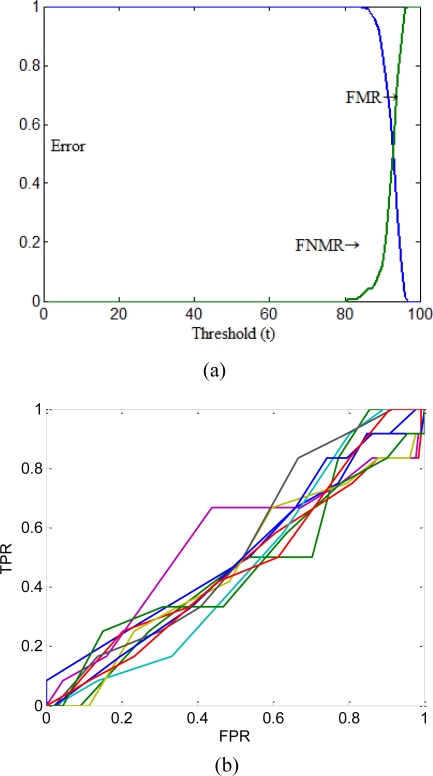
Test results for different representations (TPR: True Positive Rate, FPR: False Positive Rate). (a) FMR&FNMR representation; (b) ROC curves.

FMR&FNMR and ROC curve representations were also given in Figure 13. In the figure, ROC curves were plotted for each fold separately, but the FMR&FNMR representation curve was drawn using only average value of all folds for better comparison.

As can be seen in Figure 13, the proposed system performs the tasks with high similarity measures to the desired values. According to the numerical results given in Table 6, the proposed system was found also very successful.

**Figure 14.**
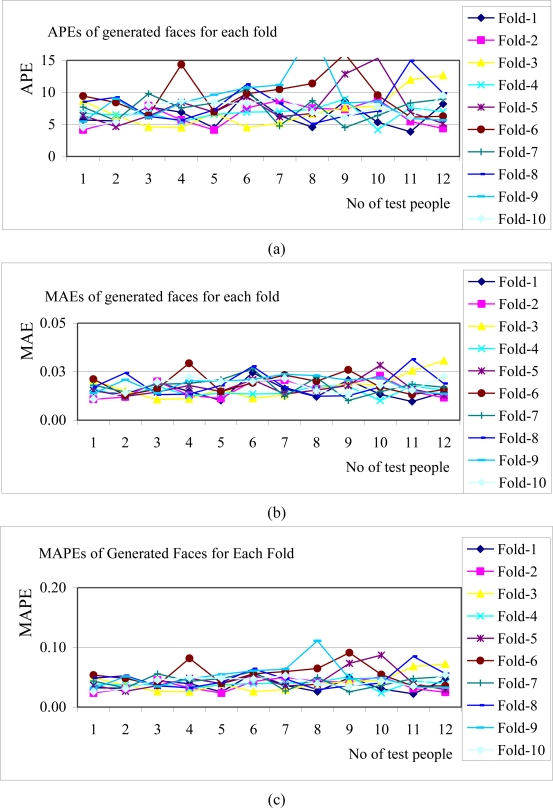
Results for APEs, MAEs and MAPEs for each fold. (a) APEs for generated faces for each fold; (b) MAEs for generated faces for each fold; (c) MAPEs for generated faces for each fold.

**Figure 15.**
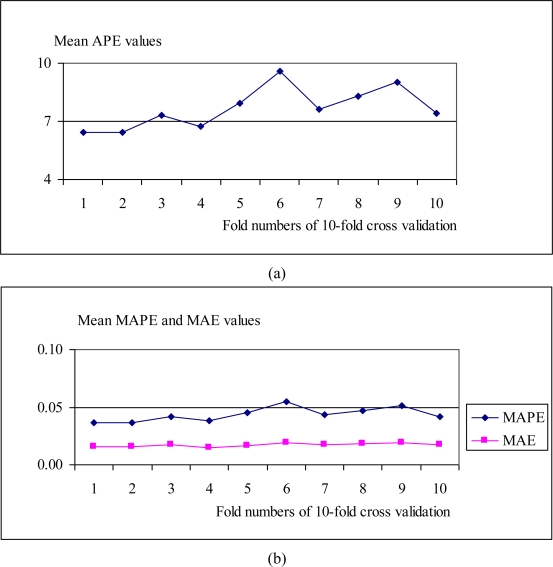
Averages of APEs, MAEs and MAPEs. (a) Averages of APE values of generated faces for each fold; (b) Averages of MAPE and MAE values of generated faces for each fold.

The APE, MAE and MAPE values belonging to all test results for each fold of 10-fold cross validation were demonstrated in Figure 14. Averages of all APEs, MAEs and MAPEs were given in Figure 15.

**Figure 16.**
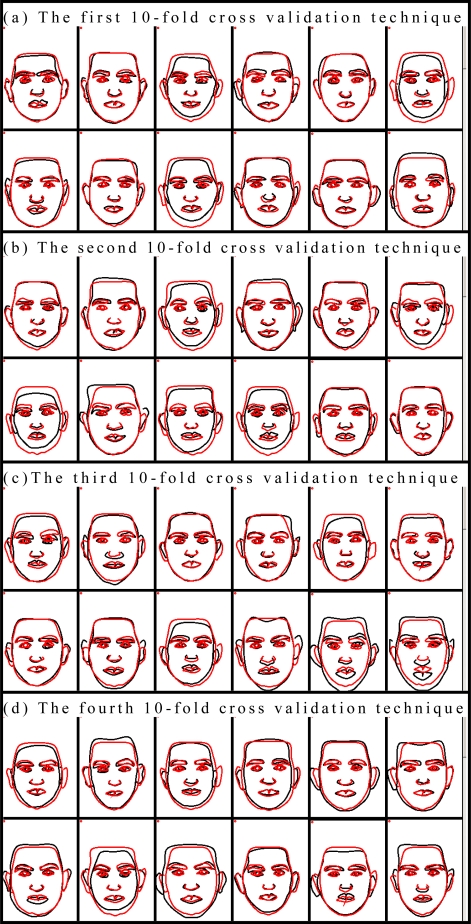

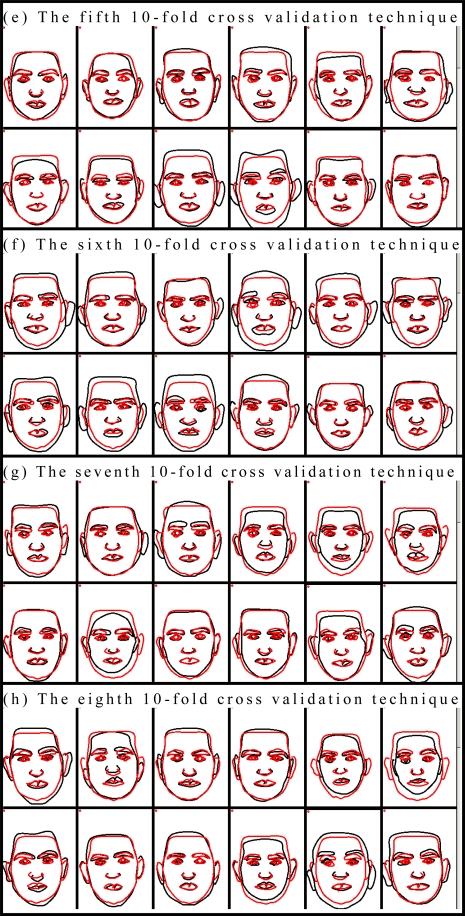

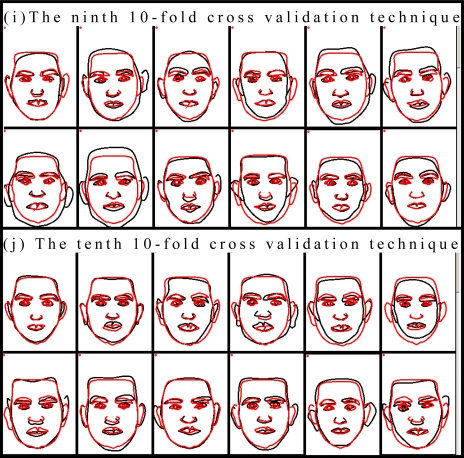
Results for 10 different test data sets.

For more realistic and comprehensive evaluation, all test results at each fold were illustrated in Figure 16 with the desired values as used in the qualitative assessment method. Dark and light lines in the figure represent the desired and the generated face features, respectively. The number of rank orders in 10-fold cross validation with 20 participants as the results of the qualitative assessment method was given in Table 7.

All observers who participated in our qualitative assessment method had normal (20/20) or corrected to normal acuity, normal color vision, and no history of ocular pathologies. In the qualitative assessment method each of the participants has assigned a numerical value of 1, 2, 3, 4 or 5 for all results of the each fold. Thus, within each condition, the system results were assigned 200 values (ten values per participant). In order to carry out a meaningful quantitative analysis, the rank frequency, that is, the number of times was assigned a rank value (*i.e.*, the number of all the ones, twos, threes, fours and fives for the results), was taken as the operational definition of perceived result quality for each fold. For each condition, the rank frequency was summed across the 10-folds, which resulted in the summed rank frequency (refer to line “Sum” in Table 7). From Table 7, it is clear that the proposed system was assigned the highest number of fives for all folds of 10-fold cross validation technique. According to the means of qualitative assessment method, the proposed system produced high quality results that were perceived to have the highest marks. Comparison for the folds of 10-fold cross validation technique can be also achieved using Table 7. According to Table 7, the first fold of the system was perceived to have the highest marks, tenth fold of the system produced imagery that was assigned the second highest number of fives (*i.e.*, essentially perceived as ‘second best’); and the seventh fold of the system produced imagery that was assigned the third highest number of fives (*i.e.*, essentially perceived as ‘third best’). For each condition the rank frequency was summed across the all folds of 10-fold cross validation technique.

Total value of the table indicates the sum of the marks for the all test results. It actually shows the overall system performance from point of the subjective manner. According to the total value, 47.5% of the participant gave 5, it means that they thought that “the results are nearly the same with the desired values, the system is”; 35.5% of the participant gave 4, it means they thought “the results are very similar to the desired values, the system is successful”, 14.5% of the participant gave 3, it also means that they thought “the results are similar to the desired values, the system success is average” and 2.5% of the participant gave 2, it means they thought “the results are a bit similar to the desired values, but the system cannot be accepted successful”. None of the participant gave 1, so no of them thought that the system is failed.

All obtained results from the two different evaluation platforms for each fold of 10-fold cross-validation technique have strongly demonstrated and clearly confirmed that there are close relationship among faces and fingerprints. Based on the results reported in this article in various forms, it can be clearly and confidently to declared that the proposed face model generation system is very successful in achieving face parts from only fingerprints. The system presented in this paper is a complete system combining all the other recent works introduced in [5–13], and it provides more complex and distinguished solution for generating the face parts. To the best of our knowledge, investigating relationships among fingerprints and face features including the all face parts has not been studied in the literature so far. Also it is the first study that was evaluated with 10-fold cross validation technique with qualitative evaluation metrics in addition to the quantitative evaluation metrics. Taguchi experimental design technique was also used to obtain best ANN parameters for better performance. Extensive experimental results have shown once more that the proposed system yields superior performance and it is capable of efficiently generating the face masks from only fingerprints.

## 9. Conclusions and Future Work

Predicting complete face features with high accuracy just from fingerprints is the principal objective of this paper. In this study a novel approach was developed, used and introduced to successfully achieve this aim. In the proposed study, the relationships among fingerprint and face biometrics were established and an unknown biometric feature was also predicted with high accuracy from a known biometric feature. The results of the two main validation tests proved that the proposed system is very successful in automatically generating the faces from only fingerprints. This study is an improved version of our earlier studies.

In the future research, investigations will be conducted to enhance the face generation process. In addition, a larger multi-modal database with international participants including Fs&Fs will be collected to investigate the proposed approach. Even if an unknown biometric feature can be achieved from a known biometric feature, the achieved feature cannot represent faces in real time face pictures. This initial study might help to lead to create new concepts, research areas, and especially new applications in the field of biometrics.

Comparing with the results given in the literature determining the best parameter settings by Taguchi experimental design technique has improved the results significantly. In addition, it should be noted that predicting more face parts from fingerprints reduced the prediction performance of the system.

For a more objective comparison, the performance and accuracy of the system have been evaluated with 10-fold cross validation technique using qualitative evaluation metrics in addition to the expanded quantitative evaluation metrics. Consequently, the results were presented on the basis of the combination of these objective and subjective metrics for illustrating the qualitative properties of the proposed methods as well as a quantitative evaluation of their performances.

## Figures and Tables

**Table 1.** The null and the alternate hypotheses for the biometric verification.

**Formulas**	**Definition**
H_o_: I≠T	Input and template are not from the same person
H_1_: I=T	Input and template are from the same person

**Table 2.** Decision types.

**Formulas**	**Definition**
D_o_: I≠T	A person is not the same person to be claimed
D_1_: I=T	A person is the same person to be claimed

**Table 3.** Two types of errors in a typical biometric system.

**Error Type**	**Formulas**	**Definition**
Type I: (FMR)	$\mathrm{FMR}=P(D_{1}|H_{0}=\mathrm{true})=\int_{t}^{1}P(s|H_{0}=\mathrm{true})\mathrm{ds}$	False match rate: (D_1_ is decided when H_o_ is true),
Type II: (FNMR)	$\mathrm{FNMR}=P(D_{0}|H_{1}=\mathrm{true})=\int_{0}^{t}P(s|H_{1}=\mathrm{true})\mathrm{ds}$	False non-match rate: (D_o_ is decided when H_1_ is true).

**Table 4.** Taguchi design factors and factor levels.

Taguchi Design		**LEVELS**							
		**1**	**2**	**3**	**4**	**5**	**6**	**7**	**8**
**DESIGN FACTORS**	Training Algorithms	CGB	CGF	CGP	GD	GDA	OSS	GDAM	SCG
	Number of Layers	3	4						
	Number of Inputs	200	300						
	Transfer Functions	HT	SF						

**Table 5.** Results for ANN Parameter Analysis.

**Factors**	Parameter Settings		
	Means	SR	Optimum Design
Training Algorithms	CGB	CGB	CGB
Numbers of Layers	3	3	3
Numbers of Inputs	300	300	300
Transfer Functions	SF	SF	SF

**Table 6.** Numerical results for comparison.

	**Maximum**	**Mean**	**Minimum**
**APE**	9.60953	7.68515	6.44791
**MSE**	0.00067	0.00038	0.00053
**SSE**	1.40740	0.79380	1.12700
**MAE**	0.01905	0.01718	0.01482
**MAPE**	0.05460	0.04367	0.03664

**Table 7.** Number of rank orders in 10-fold cross validation with 20 participants.

**No of 10-folds**	**Rank Levels**				
	**1**	**2**	**3**	**4**	**5**

The first	0	0	0	4	16
The second	0	0	2	11	7
The third	0	0	6	4	10
The fourth	0	1	3	5	11
The fifth	0	1	2	8	9
The sixth	0	3	5	10	2
The seventh	0	0	2	7	11
The eighth	0	0	4	6	10
The ninth	0	0	5	10	5
The tenth	0	0	0	6	14
Total	0	5	29	71	95
